# Regulation of Fe65 during neuronal differentiation

**DOI:** 10.1186/1750-1326-8-S1-P24

**Published:** 2013-09-13

**Authors:** Niina Koistinen, Kristin Jacobsen, Kerstin Iverfeldt

**Affiliations:** 1 Department of Neurochemistry, Stockholm University, Stockholm, Sweden

## Background

Fe65 is a ~97kDa neuronal adaptor protein which has been shown to interact with amyloid-β precursor protein (APP) and its homologues APLP1 and APLP2. Fe65 has the ability to bind to the C-terminal YENPTY motif of APP and is believed to be involved in the regulation of APP processing. APP is a type 1-transmembrane protein which can undergo regulated intramembrane proteolysis (RIP) allowing the release of a secreted ectodomain (sAPP) and a small intracellular domain called AICD. AICD has been suggested to function in regulation of gene expression by the formation of a multimeric complex with Fe65. According to previous studies retinoic acid (RA) can up-regulate the expression of both mRNA and protein levels of APP family members in SH-SY5Y cells. Because Fe65 may be essential in APP-mediated transcriptional regulation, the extent of Fe65 protein expression and its localization were investigated in RA differentiated SH-SY5Y cells. Phosphorylation of APP at specific residues has also shown to affect the processing of APP and its binding to specific adaptor proteins, such as Fe65. To further characterize how phosphorylation affects interactions between Fe65 and APP, Fe65 constructs were designed with a tandem affinity purification-tag (TAP-tag) and several Ser/Thr/ Tyr mutant constructs of myc-tagged APP was generated. APP mutants were coexpressed in SH-SY5Y cells with Fe65-TAP in order to investigate if the mutated phosphorylation sites affect the ability of Fe65 to interact with APP or if the gain/loss of interaction with Fe65 affects the processing of APP.

## Materials and methods

Human neuroblastoma SH-SY5Y cells were cultured for 6 days in the absence or presence of 10 µM all-*trans* RA. Cell- and nuclear lysates were subject to western blot analysis with antibodies against FE65. By recombinant techniques Fe65 was TAP-tagged and cloned into a pcDNA3.1(+) vector as was myc-tagged APP. Mutagenesis of APP was generated by site directed mutagenesis. Protein complexes were recovered from SH-SY5Y cell lysates by affinity purification followed by cleavage of the bound protein complex by TEV-protease. The purified Fe65-TAP protein complexes were analyzed by western blot with antibodies against Fe65 and APP.

## Results

The levels of full length FE65 was increased in the presence of RA. In addition, an electrophoretic mobility shift of the full length Fe65 was observed, possibly due to posttranslational modification. Furthermore, a nuclear ~65 kDa fragment was observed. All three members of the APP-family can be pulled-down by Fe65-TAP. Upon co-transfection of Fe65-TAP/APPwt and Fe65-TAP/APP-T668A the increased interaction of Fe65-TAP with APP seems to be blocked by the mutation.

## Conclusions

Neuronal differentiation alters levels and processing of Fe65. Furthermore, the increased Fe65-*APP* interaction seems to be dependent on APP Thr668 phosphorylation.

